# 136. Impact of an OPAT Pharmacist on Guideline Adherence and Clinical Outcomes

**DOI:** 10.1093/ofid/ofab466.136

**Published:** 2021-12-04

**Authors:** Alice Lin, Trisha S Nakasone, Nancy N Nguyen, Catherine Yang

**Affiliations:** 1 Veterans Affairs Palo Alto Health Care System, West Covina, California; 2 Veterans Affairs Palo Alto Health Care System; University of the Pacific, Thomas J. Long School of Pharmacy & Health Sciences, Palo Alto, California

## Abstract

**Background:**

Outpatient parenteral antibiotic therapy (OPAT) provides select patients a cost-effective alternative to completing intravenous (IV) antibiotic therapy outside the hospital. The Infectious Diseases Society of America (IDSA) OPAT practice guidelines and handbook recommend weekly laboratory monitoring and timely follow-up for OPAT patients. An analysis at VA Palo Alto Healthcare System (VAPAHCS) conducted prior to pharmacist involvement demonstrated that IDSA recommendations were not routinely followed, leading to a clinical cure rate of 62.7%. This led to the implementation of an OPAT pharmacist in 2019. This analysis aims to determine the impact of a pharmacist-managed OPAT program at VAPAHCS.

**Methods:**

This comparative, retrospective analysis included patients who received OPAT at VAPAHCS between October 1, 2019 and September 30, 2020 and those who received OPAT in a prior analysis. Primary outcomes included rates of adherence to IDSA recommendations on follow-up visits and weekly lab monitoring during OPAT. Secondary outcomes included rates of clinical cure, 90-day readmission, and adverse events or complications. Data was analyzed using Fisher’s exact test and independent t-test.

**Results:**

This analysis included 74 patients and 76 total OPAT episodes. Bacteremia was the most common diagnosis (n=35, 38.0%), and the most common organism was methicillin-susceptible *Staphylococcus aureus* (MSSA) (n=23, 29.9%). With respect to guideline adherence pre- and post- pharmacist-managed OPAT, 31.3% versus 93.4% of patients had follow-up within 7 to 14 days of discharge (p< 0.001). Rates of weekly lab monitoring of CBC, BMP, and LFTs pre-pharmacist were 63.2%, 63.3%, and 49.5%, respectively, compared to post-pharmacist rates of 93.0%, 92.1%, and 83.6%, respectively. Clinical cure rates were 62.7% pre-pharmacist and 89.6% post-pharmacist (p< 0.001). More adverse drug reactions were identified in the post-pharmacist period, of which 30% required pharmacist intervention.

Figure 1. Weekly Laboratory Monitoring of Antimicrobials (%)

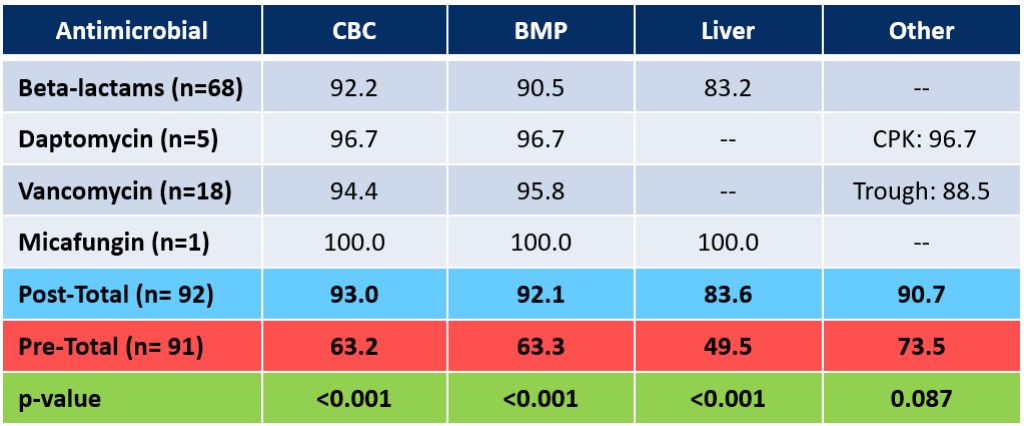

Figure 2. Adherence to IDSA Guideline Follow-up Recommendation

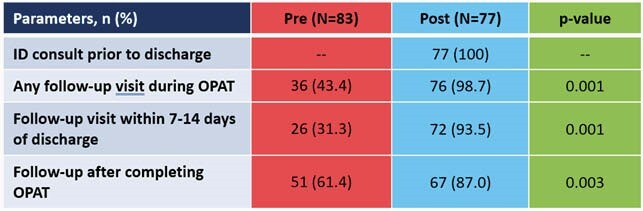

Figure 3. Rates of Clinical Cure

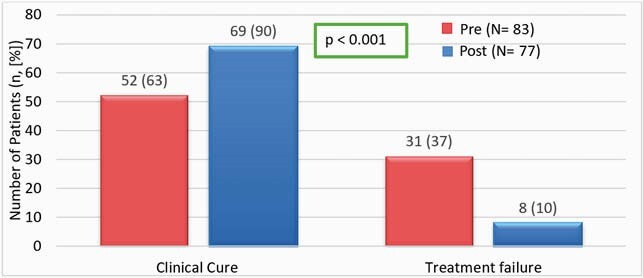

**Conclusion:**

Pharmacist involvement in OPAT significantly increased IDSA guideline adherence to lab monitoring and follow-up visits, and clinical cure rates. Identification of adverse drug reactions prompting pharmacist intervention further highlights the importance of follow-up in OPAT patients.

**Disclosures:**

**All Authors**: No reported disclosures

